# Combination of *Salvia miltiorrhiza* and ligustrazine attenuates bleomycin-induced pulmonary fibrosis in rats via modulating TNF-α and TGF-β

**DOI:** 10.1186/s13020-018-0194-9

**Published:** 2018-07-04

**Authors:** Chengliang Huang, Xu Wu, Shengpeng Wang, Wenjun Wang, Fang Guo, Yuanyuan Chen, Bi Pan, Ming Zhang, Xianming Fan

**Affiliations:** 1 Department of Respiratory Medicine II, The Affiliated Hospital of Southwest Medical University, Luzhou, Sichuan China; 2 Laboratory of Molecular Pharmacology, Department of Pharmacology, School of Pharmacy, Southwest Medical University, Luzhou, Sichuan China; 3 State Key Laboratory of Quality Research in Chinese Medicine, Institute of Chinese Medical Sciences, University of Macau, Macao, China

**Keywords:** Ligustrazine, Pulmonary fibrosis, *Salvia miltiorrhiza*, SMAD4, TNF-α

## Abstract

**Background:**

Idiopathic pulmonary fibrosis (IPF), a chronic, progressive, fibrosing interstitial lung disease, is associated with extremely poor prognosis, and lacks effective treatment. The frequently used immunosuppressive therapies such as dexamethasone (DEX) are often associated with side effects. Recently, combination of two Chinese herbal medicine preparations, *Salvia miltiorrhiza* and ligustrazine (SML), serves as an alternative medicine for treatment of IPF in clinical practices in China. The aim of this study is to compare the anti-fibrotic effect of SML with that of DEX and to investigate the underlying mechanisms.

**Methods:**

A rat model of bleomycin (BLM) induced pulmonary fibrosis was used in this study. Ninety rats were assigned to six groups: control group; BLM-group; BLM and dexamethasone group (BLM + DEX); BLM + low-dose SML; BLM + medium-dose SML and BLM + high-dose SML. Rats were sacrificed on day 7, 14 and 28 after treatment. The extent of alveolitis and fibrosis was observed by H&E and Masson’s trichrome staining. The expressions of TNF-α, TGF-β1 and SMAD4 were determined and quantified by immunohistochemical analysis. The serum levels of TNF-α and TGF-β1 were further quantified by ELISA kits.

**Results:**

Both DEX and SML treatment attenuated BLM-induced lung injury and pathological collagen deposition in rats, showing improved alveolitis and fibrosis scores on day 7, 14, 28, compared to the BLM group (*p *< 0.05). The anti-fibrotic effect of SML was in a dose-dependent manner, and the medium- and high-dose SML showed comparable effect with DEX on day 14 and 28. Expressions of TNF-α, TGF-β1 and SMAD4 were significantly decreased in the DEX- and SML-treated groups compared with BLM groups (*p *< 0.05). Medium- and high-dose SML showed better repression of TNF-α, TGF-β1 and SMAD4 expression compared to DEX at all time points (*p *< 0.05). Notably, SML at different dosages did not affect serum levels of alanine aminotransferase, aspartate aminotransferase and creatinine.

**Conclusions:**

SML is safe and effective in repressing BLM-induced pulmonary fibrosis, which might be through modulating the expression of TNF-α and TGF-β1. Our findings advocate the use of SML for IPF, which might serve as a better treatment option over DEX.

**Electronic supplementary material:**

The online version of this article (10.1186/s13020-018-0194-9) contains supplementary material, which is available to authorized users.

## Background

Idiopathic pulmonary fibrosis (IPF) is a chronic, progressive, fibrosing interstitial lung disease characterized by alveolar epithelial cell injury, aberrant proliferation of fibroblasts and excessive extracellular matrix (ECM) deposition leading to progressive fibrosis and loss of lung function, which accounts for about 20% of all cases of interstitial lung disease [1]. IPF is the most common and severe among the idiopathic interstitial pneumonias [2] and is associated with extremely poor prognosis with median survival time of 2–3 years [3]. Several risk and predisposing factors, including cigarette smoking, viral infections, gastro-oesophageal reflux and surfactant protein polymorphisms, have been reported to contribute to the pathogenesis of IPF [4, 5]. Although the pathological mechanisms remain unclear, increasing evidences demonstrate that repetitive injury to the alveolar epithelial cells drives aberrant wound healing responses, resulting in fibrosis [1, 6]. Several cytokines and chemokines have been reported to involved in this process [5, 6]. Among them, TGF-β, the major profibrotic mediator, plays central role in the development of fibrosis by inducing differentiation of lung fibroblasts into myofibroblasts, stimulating ECM accumulation and epithelial mesenchymal transition (EMT) [7, 8]; TNF-α, the cytokine with inflammatory and fibrotic properties, stimulates collagen synthesis, induces TGF-β and promotes proliferation of fibroblasts [8, 9]. Elevated levels of TGF-β and TNF-α have been found in the lungs of animals in experimental models of pulmonary fibrosis and in patients with IPF [10, 11]. Blocking of TNF-α and TGF-β signalling resulted in attenuation of fibrosis in rodents [12, 13], which served as a promising therapeutic strategy for IPF.

Currently, lung transplantation is the only effective treatment for IPF. The frequently used immunosuppressive therapies such as dexamethasone are often associated with side effects and have not been proven to improve the survival and quality of life of the patients [14]. Notably, the combination of two Chinese herbal medicine preparations, *Salvia miltiorrhiza* (Danshen) injection and ligustrazine injection, has been widely used for treatment of IPF in clinical practices, showing good efficacy and few side effects [15, 16]. Danshen, the root and rhizome of *Salvia miltiorrhiza* Bge., is widely used in traditional Chinese medicine for the treatment of various ailments such as those affecting the circulatory, hepatic and respiratory systems [17]. Recent studies has shown that *Salvia miltiorrhiza* and its main constituents dramatically attenuated pulmonary fibrosis in mice models through reducing inflammation [18, 19]. Furthermore, ligustrazine, the main active ingredient of *Ligusticum chuanxiong* hort (Chuanxiong), has been proved to have protective effects against lung fibrosis induced by hyperoxia and bleomycin [20, 21]. The pair of *Salvia miltiorrhiza* and ligustrazine has been commercially available in China as a compound preparation, namely Danshen–ligustrazine injection. Despite the potential in treating IPF, the mechanisms underlying the actions of combination of *Salvia miltiorrhiza* and ligustrazine (SML) in pulmonary fibrosis are not fully elucidated. In the present study, we aimed to compare the anti-pulmonary fibrosis effects of SML with that of DEX in a rat model of bleomycin-induced pulmonary fibrosis and to investigate the underlying mechanisms.

## Methods

The minimum standards of reporting checklist (Additional file 1) contains details of the experimental design, and statistics, and resources used in this study.

### Chemicals and reagents

Bleomycin (BLM-A5) was purchased from Tianjin Taihe Pharmaceuticals Ltd (Tianjin, China). *Salvia miltiorrhiza* injection, the standard aqueous extract of *Salvia miltiorrhiza*, was purchased from Sichuan Sanjing Shenghe Pharmaceuticals Ltd. (Sichuan, China). Ligustrazine injection was purchased from Henan Furen Pharmaceuticals Ltd. (Henan, China). The immunohistochemistry kits used for the detection of TNF-α, TGF-β1 and SMAD4 were purchased from Dako (cat. no. K500711). ELISA kits for detection of serum TNF-α and TGF-β1 were obtained from MultiSciences (China). All other chemicals and reagents used were of analytical grade or of the highest grade available.

### Animals

Ninety male Sprague–Dawley rats weighing about 180–220 g (6–7 weeks) were obtained from the animal center of Southwest Medical University (Luzhou, China). Animals were housed 2–3 per cage in a controlled environment (temperature, 25 ± 2 °C; humidity, 30–70%) with an alternating 12 h light/dark photoperiod. The rats were acclimatized for a week before the start of the experiment and fed with standard rodent chow and water ad libitum. The study was approved by Southwest Medical University Animal Ethics Committee and conducted in accordance with the Guide for the Care and Use of Laboratory Animals [22].

### Study design and treatment protocol

After recording the body weights, the rats were anesthetized with an intraperitoneal injection of ketamine hydrochloride (80 mg/kg, b.wt) and xylazine (20 mg/kg, b.wt). A midline incision was made in the neck and the trachea was exposed. A tracheal cannula was inserted under direct visualization, and a single intratracheal instillation of bleomycin BLM-A5 (5 mg/kg, b.wt) dissolved in sterile 0.9% NaCl was delivered on day 1 of the experiment for induction of pulmonary fibrosis as previously reported [23].

Rats were randomly allocated into six groups with fifteen animals in each group. Group I animals received intratracheal injection of physiological saline alone (0.2–0.3 mL) and served as control group; Group II animals were subjected to a single intratracheal instillation of BLM (5 mg/kg b.wt in sterile 0.9% NaCl); Group III animals received BLM in the same as group II and treated with DEX (1.4 mg/kg, i.v. injection via tail vein); Group IV animals received BLM in the same as group II along with low-dose SML (SM + L: 125 + 43.75 mg/kg, i.v. injection via tail vein); Group V animals received BLM as group II and supplemented with medium-dose SML (SM + L: 250 + 87.5 mg/kg, i.v. injection via tail vein); Group VI received BLM as group II and supplemented with high-dose SML (SM + L: 500 + 175 mg/kg, i.v. injection via tail vein). Rats in each group was further divided into three subgroups and were sacrificed on day 7, 14, and 28 after BLM instillation, respectively.

### H&E and Masson’s trichrome staining

The left lungs of rats were excised, fixed in 10% neutral buffered formalin and embedded in paraffin. The paraffin-embedded tissue samples were sectioned into 5-μm slices, then deparaffinized, and stained with H&E as well as Masson’s trichrome to investigate the degree of lung tissue inflammation and fibrosis, respectively. The sections were examined and evaluated randomly using standard light microscopy (DP73, Olympus) by an experienced histologist, unaware of the treatment groups. The pulmonary fibrosis grade was scored by examining 10 randomly selected regions per sample at 200× magnification for fibrotic tissue and collagen and the scores were averaged per group. The criteria used for grading lung fibrosis were as follows: Grade 0, normal; Grade 1, minimal fibrous thickening of alveolar or bronchial walls; Grades 2–3, moderate thickening of walls without obvious lung damage; Grades 4–5, increased fibrosis with definite lung damage and formation of fibrous bands or small fibrous mass; Grades 6–7, severe distortion of structure and large fibrous areas or “honeycomb lung”; and Grade 8, total fibrous obliteration of the field [9, 23].

### Immunohistochemical analysis

The lung tissue sections were deparaffinized in xylene and rehydrated using graded ethanol solutions. The endogenous peroxidase activity was quenched using 0.3% hydrogen peroxide in methanol for 15 min. To eliminate non-specific binding, the sections were blocked with 5% BSA, 0.1% Tween-20 in TBS and incubated for 1 h. Following blocking, the sections were rinsed with TBST buffer containing 0.1% Tween-20. The tissue sections were incubated with TNF-α, TGF-β1 and SMAD4 polyclonal antibodies (1:100 for both) overnight at 4 °C. The sections were re-equilibrated to room temperature and washed with TBS, followed by the incubation with streptavidin–biotin–peroxidase conjugated secondary antibody for 1 h at room temperature (Dako LSAB). Finally, reaction products were visualized with 3,3′-diaminobenzidine (DAB) and then counterstained with haematoxylin. Images were collected using the inversion fluorescence microscope (Leica DMIRB) and computer image acquisition software (Leica). The Graphic context analysis software (ImagePro plus 6.0) was used to analyze the pictures of immunohistochemistry. Five fields were chosen under the 200× microscope for each slice to record the positive staining for average integral optical density.

### Quantification of serum TNF-α and TGF-β1 levels

The serum levels of TNF-α and TGF-β1 were determined using ELISA kits following the manufacturer’s instruction.

### Biochemical analysis of rat serum

On day 28th, rats were anaesthetized and blood were collected through cardiac punctuation. Serum were obtained for determination of alanine aminotransferase (ALT), aspartate aminotransferase (AST) and creatinine (Cre) on an Advia 2400 Clinical Chemistry System (Siemens Healthcare).

### Statistical analysis

All the results were expressed as mean ± SD. Statistical analysis was performed with Graphpad Prism 7.0 using one-way or two-way ANOVA followed by Tukey’s multiple comparisons test. The *p* value < 0.05 was considered as statistically significant.

## Results

### SML attenuated BLM-induced lung injury and inflammation

**Fig. 1:**
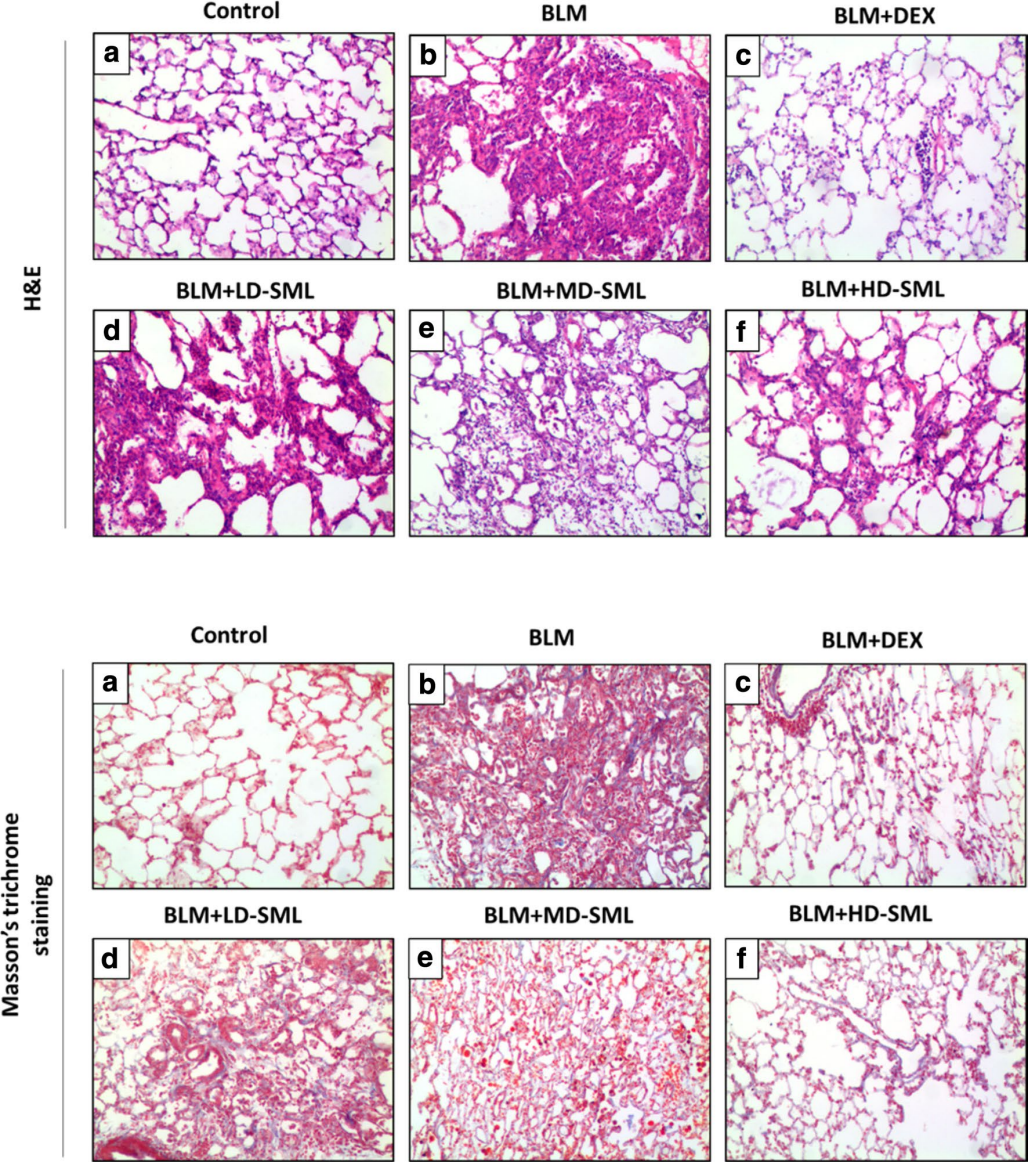
Representative photographs for H&E (upper panel) and Masson’s trichrome (lower panel) staining of rat lung tissues on the 28th day. H&E staining of lung tissue sections are shown in the upper panel: **a** control animals showing normal lung tissue morphology; **b** bleomycin treated animals showing distorted lung morphologies with inflammatory cell infiltration, thickened interalveolar septa, alveolar edema, alveolar exudate, collapsed alveolar spaces with inflammatory exudates; **c** dexamethasone treated rats with significant less inflammatory cell infiltration; **d**–**f** animals treated with low-dose (LD), medium-dose (MD) and high-dose (HD) SML showing less inflammation compared to bleomycin alone treated animals. Masson’s trichrome staining for collagen assessment in lung tissues are shown in the lower panel: **a** lung tissue sections of control animals showing scarcely deposited collagen in the lung parenchyma; **b** BLM-induced animals showing dense collagen accumulations; **c** DEX-treated animals showing less collagen deposition as compared to BLM-induced group; **d**–**f** low-dose (LD), medium-dose (MD) and high-dose (HD) SML-treated animals showing improved collagen deposition compared to BLM-induced group

The histopathological alterations using H&E staining in different groups are depicted in Fig. 1a. The lung tissue sections of control rats showed normal architecture with intact alveolar epithelium, normal thickening of alveolar septa and no pathological changes such as alveolitis or interstitial pulmonary fibrosis (Fig. 1a, upper panel). Intratracheal administration of BLM led to abnormal lung morphologies, including significant interstitial infiltration of inflammatory cells, alveolar septal thickening and collapsed alveolar spaces (Fig. 1b, upper panel). Notably, treatment with dexamethasone or SML prominently reduced the lung damage induced by BLM, and the effect of SML was in a dose-dependent manner (Fig. 1c–f, upper panel). The low-dose SML treatment slightly ameliorated the BLM-induced pathological changes, while the medium- and high-dose SML significantly alleviated the lung damage as shown by decrease in infiltration of inflammatory cells and thin lined alveolar septa compared with BLM-induced animals (Fig. 1f, upper panel).

**Fig. 2:**
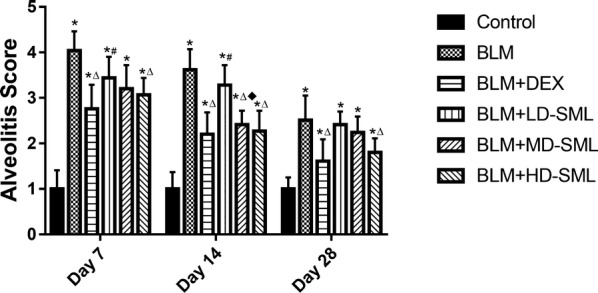
Inflammatory (alveolitis) score in the control and experimental animals at 7, 14 and 28 days of treatment. Each value is expressed as mean ± S.D. (n = 5). Results are statistically significance at *p *< 0.05. *p < 0.05 vs control; ^∆^*p* < 0.05 vs BLM; ^#^*p* < 0.05 vs BLM + DEX; ^■^*p* < 0.05 vs BLM + LD-SML, by two-way ANOVA followed by Tukey’s multiple comparisons test. *LD* low-dose, *MD* medium-dose, *HD* high-dose

Alveolitis scoring was performed depending on the severity of inflammation. In BLM group, significant infiltration (*p* < 0.05) of inflammatory cells was observed on day 7 compared to control group, which gradually subsided on day 14 and 28. Whereas, in groups supplemented with medium- and high-dose SML, significant decrease (*p* < 0.05) in the infiltration of inflammatory cells were observed in the lung tissue on day 14 and 28 as demonstrated by lower alveolitis score compared to BLM-treated rats (Fig. 2). The low-dose SML slightly ameliorated the infiltration, but the value was not significant compared to that of BLM-group at day 14 and 28. It should be noted that the medium- and high-dose SML intervention showed comparable anti-fibrotic effect compared with the DEX treatment on day 14 and 28 according to the histological examination and alveolitis scores (Fig. 1, upper panel and Fig. 2).

### SML reduced pathological collagen deposition in BLM-induced lung fibrosis

**Fig. 3:**
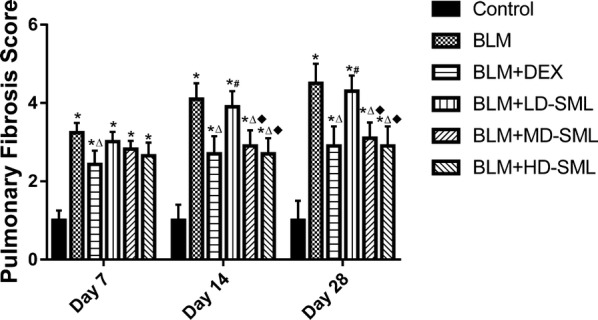
Quantitative evaluation of fibrotic changes (fibrotic tissue and collagen) in the lung sections by Ashcroft scoring at 7, 14 and 28 days of treatment. Each value is expressed as mean ± S.D. (n = 5). Results are statistically significance at *p *< 0.05. *p < 0.05 vs control; ^∆^*p* < 0.05 vs BLM; ^#^*p* < 0.05 vs BLM + DEX; ^■^*p* < 0.05 vs BLM + LD-SML, by two-way ANOVA followed by Tukey’s multiple comparisons test. *LD* low-dose, *MD* medium-dose, *HD* high-dose

Fibrillar collagen deposition, an indicator of lung fibrosis, was determined by Masson’s trichome staining (Fig. 1, lower panel). Injection of BLM increased collagen accumulation in the interstitial lung spaces with almost complete destruction of the alveolar architecture (Fig. 1b, lower panel). The collagen deposition was attenuated by administration of DEX or SML (Fig. 1c–f, lower panel), which showed fewer and smaller fibrotic foci. The Ashcroft quantitative pathological scoring of Masson’s trichrome staining is presented in Fig. 3. The fibrosis score was significantly higher (*p* < 0.05) in BLM-induced fibrotic rats compared with the negative control group at day 7, 14 and 28, while treatment of DEX or SML (medium and high-dose) significantly reduced the fibrosis score (*p* < 0.05). The low-dose SML showed no obvious anti-fibrotic effect. Of particular note, DEX and medium- or high-dose SML showed comparable extent of anti-fibrotic effect on day 14 and 28. These data supported the view that SML played an important role in reducing pathological collagen deposition and structural damage in the BLM-induced rat lung fibrosis model.

### SML represses the expression of TNF-α, TGF-β1 and SMAD4 proteins in BLM-induced lung fibrosis

**Fig. 4:**
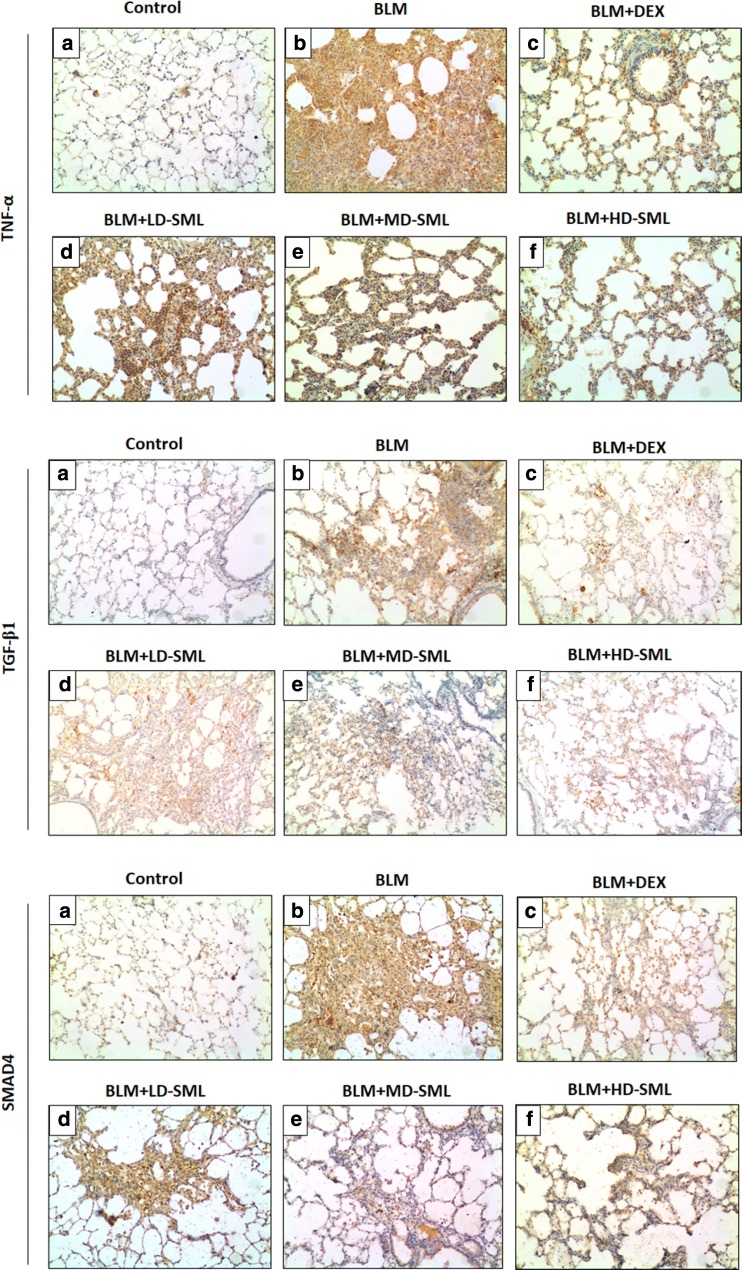
Immunohistochemical staining of TNF-α (upper panel), TGF-β1 (middle panel) and SMAD4 (lower panel) protein on the 28th day in the rat lung tissue of control and experimental animals. **a** Lung section of control group showed a small degree of immunostaining of TNF-α, TGF-β1 and SMAD4. **b** BLM-group showed increased expression of TNF-α, TGF-β1 and SMAD4 as illustrated by the brown color. **c**–**f** Sections of lung tissues from the rats treated with DEX or different doses of SML [low-dose (LD), medium-dose (MD), high-dose(HD)] showing moderate to mild positive immunoreaction of TNF-α, TGF-β1 and SMAD4

**Fig. 5:**
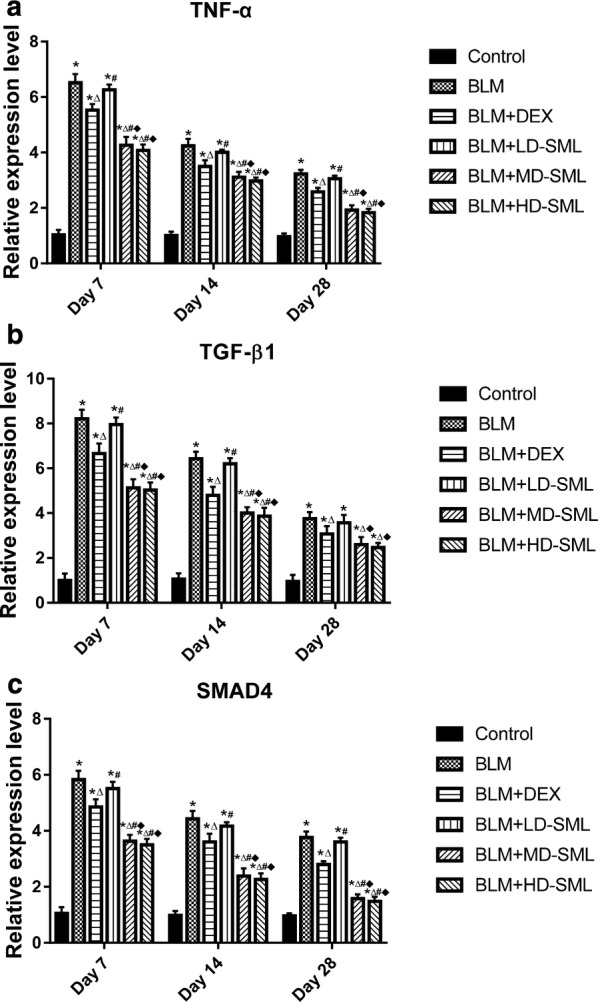
The relative expression level of TNF-α (**a**), TGF-β1 (**b**) and SMAD4 (**c**) in different groups by immunohistochemical staining on day 7, 14 and 28. The expression levels of TNF-α, TGF-β1 and SMAD4 were assessed by Graphic context analysis software (ImagePro plus 6.0). All data are expressed as mean ±S.D (n = 5). Results are statistically significance at *p *< 0.05. *p < 0.05 vs control; ^∆^*p* < 0.05 vs BLM; ^#^*p* < 0.05 vs BLM + DEX; ^■^*p* < 0.05 vs BLM + LD-SML, by two-way ANOVA followed by Tukey’s multiple comparisons test. *LD* low-dose, *MD* medium-dose, *HD* high-dose

To demonstrate the anti-inflammatory and anti-fibrotic activity of SML, the levels of TNF-α, TGF-β1 and SMAD-4 were studied in different groups by immunohistochemical analysis. SMAD-4 plays an important role in the modulation of the TGF-β pathway. The results are displayed in Figs. 4 and 5. The lung tissue from the control rats showed less expression of TNF-α, TGF-β1 and SMAD4 compared with other groups. The expression of TNF-α, TGF-β1 and SMAD4 in the DEX group and low-, medium-, high-dose SML groups were significantly reduced compared with that in BLM-group (Fig. 4). Importantly, compared with DEX group, medium and high-dose SML showed more significantly reduced expression of TNF-α, TGF-β1and SMAD4 on day 7, 14 and 28 (Fig. 5; *p* < 0.05). In particular, on day 28, the relative expression of TNF-α was significantly reduced to 1.92 ± 0.17, and 1.83 ± 0.13 in medium-, and high-dose SML groups, respectively, while that in DEX group was 2.58 ± 0.14. Similarly, the relative expression of TGF-β1 protein in medium- and high-dose SML groups on day 28 was significantly reduced to 2.59 ± 0.33, and 2.46 ± 0.20, respectively, while that in DEX group was 3.07 ± 0.35. Moreover, compared to low-dose SML, treatment with medium- and high-dose of SML showed significant decrease in the expression of TNF-α, TGF-β1 and SMAD4 (*p* < 0.05). There is no significant difference between medium- and high-dose SML in the level of TNF-α, TGF-β1 and SMAD4 protein (Fig. 5).

**Fig. 6:**
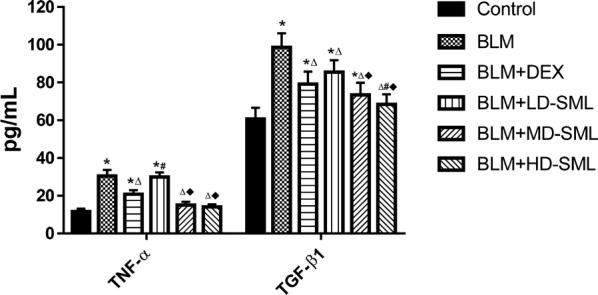
The serum levels (pg/mL) of TNF-α and TGF-β1 in different groups by ELISA on day 28. All data are expressed as mean ± S.D. (n = 5). Results are statistically significance at *p *< 0.05. *p < 0.05 vs control; ^∆^*p* < 0.05 vs BLM; ^#^*p* < 0.05 vs BLM + DEX; ^■^*p* < 0.05 vs BLM + LD-SML, by one-way ANOVA followed by Tukey’s multiple comparisons test. *LD* low-dose, *MD* medium-dose, *HD* high-dose

The above results were further confirmed by quantitatively analysis of serum levels of TNF-α and TGF-β1 using ELISA kit. The data shown in Fig. 6 indicated that DEX and medium- and high-dose SML significantly decreased the serum concentrations of TNF-α and TGF-β1 at day 28 (*p* < 0.05). The medium- and/or high-dose SML showed a better effect than DEX to repress TNF-α and TGF-β1. Moreover, compared to the serum levels of TNF-α and TGF-β1 in medium- and high-dose of SML groups were lower than that in low-dose SML group (*p* < 0.05). These results indicated that SML treatment attenuated BLM-induced fibrosis via significantly reducing the expression of TNF-α and TGF-β1 in fibrotic lung tissues.

### SML did not induce toxicity in rats

**Fig. 7:**
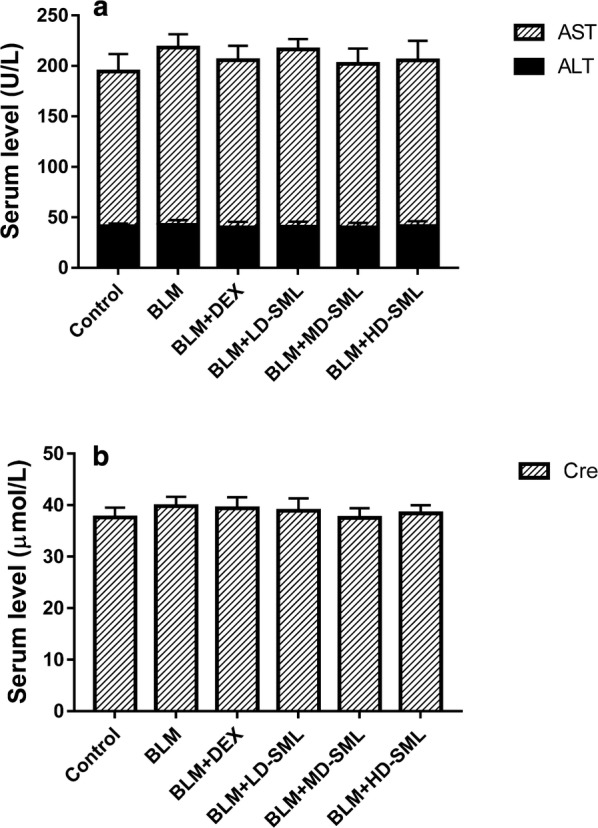
The serum levels of ALT, AST and Cre in rats on day 28th. All data are expressed as mean ±S.D. (n = 5). Statistical analysis was performed by one-way ANOVA followed by Tukey’s multiple comparisons test

The serum levels of ALT, AST and Cre in rats on day 28th were determined to assess the safety of applying SML and DEX. ALT and AST are important indices commonly used for evaluating liver function, while Cre is one of the most critical indices to monitor kidney function. The results (Fig. 7) indicated that the serum levels of ALT, AST and Cre in all rats were within the normal physiological range, and no differences were found among different groups. This suggested that SML and DEX at the designed dosage did not induce significant toxicity in rats.

## Discussion

IPF is a progressive fatal lung disease characterized by epithelial/fibroblastic disarray with excessive ECM deposition, which is due to injury to the alveolar epithelial cell, re-modelling of the ECM matrix, proliferation and accumulation of fibroblast, resulting in impaired lung function and death. Currently, there is no complete cure for IPF, and the treatment options available are limited. This necessitates the need for developing new modalities for treating this devastating disorder. In this study, the anti-fibrotic role of SML against BLM-induced pulmonary fibrosis was evaluated. We showed evidence that SML prevented BLM-induced fibrosis by attenuating inflammation and collagen deposition and modulating the expression of TNF-α and SMAD4.

BLM is a chemotherapeutic agent used in the management of various neoplastic diseases such as lymphomas, head and neck squamous cell carcinomas, testicular carcinomas, ovarian cancer and malignant pleural effusions [24]. The development of pulmonary fibrosis is the major dose-limiting side effect of this anticancer agent. Various methods to induce lung damage similar to human IPF are available by using chemicals (BLM, peplomycin, fluorescein isothiocyanate, vanadium pentoxide, trinitrobenzene sulfonic acid), growth factor gene over-expression (TGF-β, IL-1β, IL-13, etc.) and inorganic particles (silica, asbestos). Amongst all, BLM-induced pulmonary fibrosis is ideal, reproducible, consistent. The established animal model, as intratracheal instillation, mimics all the clinicopathological features of human IPF [25]. BLM-induced pulmonary fibrosis consists of two phases. The first phase is characterized by predominant inflammatory component where activated inflammatory cells release increased amounts of ROS and RNS resulting in parenchymal injury, and this occurs in 2-weeks after BLM administration. The inflammatory phase is followed by late fibrotic phase between the 3rd and 4th week, and is characterized by intense deposition of ECM, resulting in fibrosis [24, 26]. In the present study, BLM caused distortion of lung morphology with interstitial infiltration of inflammatory cells, peribronchial and perialveolar septal thickening and collapsed alveolar spaces (Fig. 1, upper panel). Moreover, BLM administration increased collagen accumulation in the interstitial lung spaces. Treatment with DEX and SML significantly reduced the damage and attenuated the collagen deposition induced by BLM demonstrating the protective effect in BLM-induced fibrosis. This is also evident from the decreased alveolitis and fibrosis scores in SML-treated animals compared to BLM-group.

Cytokines play an important role in pathogenesis of pulmonary fibrosis [27]. Different cytokines interact with each other and forms a complicated network which play the key role in the pathogenesis of IPF. Many researchers investigated the effect of TNF-α in IPF, and found that TNF-α plays an important role in the development of interstitial inflammation and pulmonary fibrosis. TNF-α regulates the apoptosis of respiratory epithelial cells, induces the expression of other cytokines and inflammatory mediators [28]. Also, TNF-α up-regulates the expression of TGF-β1, activates NF-κB, and promotes the proliferation, differentiation of fibroblasts finally induces pulmonary fibrosis [8, 24]. Previous studies have demonstrated that infliximab, a TNF-α antagonist, improved the degree of pulmonary fibrosis induced by BLM in rats [12]. Moreover, a randomized, placebo-controlled, multicentred trial demonstrated that etanercept, another TNF-α antagonist, decreased the rate of disease progression in IPF patients [10]. In the present study, increased expression of TNF-α was observed in the lung tissues of BLM-treated rats. Treatment of SML attenuated the expression of TNF-α. Importantly, the medium- and high-dose SML showed much better activity than DEX on days 7, 14 and 28.

TGF-β, the pro-fibrotic cytokine, plays a key role in pulmonary fibrosis by inducing fibroblast activation, myofibroblast differentiation with increased αSMA expression and ECM accumulation. TGF-β1 signalling from the cell membrane to the nucleus occurs mainly via SMAD proteins. SMAD4 combines with the activated SMAD2/3 to form a tripolymer, which activates the promoter of ECM, like collagen, in the cell nucleus. This might be the process which explains the involvement of SMAD4 in the occurrence and progression of pulmonary fibrosis. Recent study has shown that pirfenidone, the anti-fibrotic agent, significantly repressed the TGF-β1 induced expression of SMAD2/4 proteins and prevented the nuclear accumulation and translocation of SMAD2 protein, thereby inhibiting proliferation, migration and differentiation of TGF-β1-stimulated murine mesenchymal stem cells [29]. In the present study, SML reduced the expression of both TGF-β1 and SMAD4. Similar to SML, many natural compounds including berberine [13], daidzein [30], and ginsenoside Rg1 [31] exerted protective effect by intervening the TGF-β/SMAD signalling pathway. The advantage of using natural products, including SML, is that they usually do not induce significant side effects in body even with a long-term application. In our study, it is found that both SML did not induce toxicity in rats, as evidenced by no significant changes in ALT, AST and Cre levels in serum. However, DEX, the typical immunosuppressive therapy, always leads to unwanted adverse effects on cardiac, digestive and skin systems. Taken together, in the present study, SML decreased the tissue expression of TNF-α and TGF-β1, and improved pulmonary fibrosis in rats, which was much effective at medium and high doses compared with DEX. Hence, the current study confirms that SML exhibits anti-fibrotic activity by repressing the expression of TNF-α and TGF-β1 proteins.

## Conclusions

The present study demonstrated the anti-fibrotic effect of SML in BLM-induced pulmonary fibrosis as evidenced from the H&E and Masson’s trichome staining, showing improved alveolitis and fibrosis scores. Furthermore, SML alleviates BLM-induced fibrosis by decreasing the expression of TNF-α, TGF-β1 and SMAD4 in the lung tissues. We also compared the anti-fibrotic effect of SML with DEX, a drug commonly used for IPF. SML at the medium or high dose showed comparable or even higher extent of anti-fibrotic effect with that of DEX. These findings reveal the beneficial effect of SML against BLM mediated fibrotic challenge through suppressing TNF-α and TGF-β mediated fibrotic events. However, further research is warranted to elucidate the mechanisms regarding SML regulation on the profound cellular events of BLM-induced pulmonary fibrosis.

## Additional file

**Additional file 1.** Minimum Standards of Reporting Checklist.

## Abbreviations

ALT: alanine aminotransferase
AST: aspartate aminotransferase
BLM: bleomycin
Cre: creatinine
DEX: dexamethasone
ECM: excessive extracellular matrix
IPF: idiopathic pulmonary fibrosis
SML: combination of *Salvia miltiorrhiza* and ligustrazine
